# Atypical Antipsychotics as Augmentation Therapy in Anorexia Nervosa

**DOI:** 10.1371/journal.pone.0125569

**Published:** 2015-04-29

**Authors:** Enrica Marzola, Nadia Desedime, Cristina Giovannone, Federico Amianto, Secondo Fassino, Giovanni Abbate-Daga

**Affiliations:** Eating Disorders Center for Treatment and Research, Department of Neuroscience, University of Turin, Turin, Italy; University of Western Brittany, FRANCE

## Abstract

Anorexia nervosa (AN) is a life-threatening and difficult to treat mental illness with the highest mortality rates of any psychiatric disorder. We aimed to garner preliminary data on the real-world use of olanzapine and aripiprazole as augmentation agents of Selective Serotonin Reuptake Inhibitors (SSRIs) in adult inpatients affected by AN. We retrospectively evaluated the clinical charts of patients who were hospitalized between 2012 and 2014. Patients were evaluated upon admission and discharge. We investigated eating symptomatology, and both general and eating psychopathology using: Hamilton Rating Scale for Anxiety, Hamilton Rating Scale for Depression, and Yale-Brown-Cornell Eating Disorders Scale. The charts of 75 patients were included in this study. The sample resulted equally distributed among those receiving SSRIs and either aripiprazole or olanzapine in addition to SSRIs. Notwithstanding a few baseline clinical differences, upon discharge all groups were significantly improved on all measures. Interestingly, aripiprazole showed the greatest effectiveness in reducing eating-related preoccupations and rituals with a large effect size. The body of evidence on medication management in AN is in dismal condition. Augmentation therapy is a well-established approach to a variety of mental disorders and it is often used in every-day clinical practice with patients affected by AN as well. Nevertheless, to date very little data is available on this topic. Results from our sample yielded promising results on the effectiveness of aripiprazole augmentation in reducing eating-related obsessions and compulsions. Randomized controlled trials are warranted to confirm these encouraging findings.

## Introduction

AN is a severe mental disorder with a relevant biological predisposition whose etiology is complex and still largely unknown [1]. The course of AN is often relapsing and in a substantial proportion of cases an enduring and treatment-resistant disorder occurs [2]. However, over the past decades new insights into the neurobiology of this disorder emerged. In particular, several lines of research have shed light on the imbalances of serotonin [3] and dopamine [4] systems in AN with the former potentially being involved in altered satiety and mood and the latter in altered reward with respect to food and motivation [5].

No proven effective treatments, including pharmacotherapy, are currently available for patients affected by AN [6] and the difficulties in performing large-scale randomized controlled trials (RCTs) in this research field have been widely acknowledged [7]. Earlier studies showed that first-generation antipsychotics should be used with caution to treat AN because of short- and long-term side effects [8]. Nevertheless, over the last years increasing interest has been devoted to the use of atypical antipsychotics (AAs) in the treatment of AN (for reviews see [9–12]). The rationale for using atypical antipsychotics in AN is grounded on: a) the neurobiology of AN, with the alterations of dopamine and serotonin pathways in the brain [3–5]; b) the antidopaminergic properties of these medications that could mitigate sufferers’ obsessional thinking towards weight and body shape [9]; c) AA positive effects on safety, anxiety, eating psychopathology [9] and depression [11]; d) the increase in appetite and food intake that AA entail, consequently enhancing weight restoration, given the high-affinity profile to serotonergic, histaminergic, and adrenergic receptors [9]. A handful of case reports and open trials described the use of quetiapine [13–15], amisulpride [16], and aripiprazole [17] for adult patients diagnosed with AN. Controlled trials investigated the effectiveness of olanzapine in adult patients with AN [18–20] providing mixed results with respect to weight gain but overall supporting the effectiveness of this AA on patients’ comorbid conditions like depression, anxiety, and obsessive-compulsive traits. Nevertheless, recent meta-analysis [9,11,12] have called into question the effectiveness of AA medications, although their usefulness for subgroups of patients cannot be ruled out [9]. In fact, the modest number of available RCTs makes it difficult to ascertain whether specific subgroups of patients might benefit from using AA and an individualized clinical judgment should guide the treatment choice [9].

Converging evidence indicates that patients affected by AN are frequently characterized by comorbid disorders, mainly anxiety disorders, obsessive-compulsive disorder, and major depressive disorder [21,22]. Notwithstanding this overlap and some encouraging findings [23,24], antidepressants failed to be effective in clinical trials in AN [25] and their impact on depressive comorbidity has been recently questioned [26].

Surprisingly, evidence is still lacking as regards the combination of SSRIs and AAs. This is noteworthy in the light of a couple of considerations. Firstly, AAs have been widely used since decades in general psychiatry as augmentation agents for severe forms of depression and obsessive features [27,28]. Secondly, on one hand the association of different medications is common in clinical practice in AN [17] but on the other hand such data are very difficult to quantify and report.

Given the aforementioned gaps in literature, with this retrospective study we aimed to garner preliminary data on the real-world use of AAs as augmentation agents of SSRIs in AN. Our research question focused on olanzapine and aripiprazole with the former being included on the basis of the aforementioned literature [9, 18–20]. Aripiprazole was selected in an exploratory fashion because of a twofold rationale: a) its beneficial effects suggested not only by our clinical experience but also by authoritative groups in the AN field [17]; b) the dearth of data on its use in AN in spite of its efficacy on frequently reported comorbid conditions in psychiatric patients [28, 29]. We hypothesized that the augmentation of SSRIs with AAs could be more effective than SSRI monotherapy particularly with respect to depression and obsessive-compulsive features, in line with other fields in psychiatry [28,29]. In fact, depressive symptomatology is a frequent comorbid condition in AN, mostly for those who are hospitalized [30] as well as the presence of obsessive traits, either eating-related or not [21,31]. Moreover, both comorbid conditions can impact on patients’ engagement in treatment [32]. We expected to find olanzapine as more effective than other medications on weight gain [18,20], and aripiprazole and olanzapine as equally effective on obsessive-compulsive aspects [28] of AN.

## Materials and Methods

### Participants

We retrospectively evaluated the clinical charts of patients who were hospitalized between January 2012 and May 2014 at the ward for Eating Disorders of the San Giovanni Battista Hospital of the University of Turin, Italy. To be included, patients had to meet full criteria for AN both subtypes according to DSM-IV-TR [33] as assessed using the Structured Clinical Interview for DSM-IV Axis I Disorders (SCID-I) [34]. In addition, patients had to be already on an SSRI upon admittance for at least 6 weeks and either olanzapine or aripiprazole had to be added as augmentation therapy during hospitalization. Low-doses of benzodiazepines did not represent an exclusion criterion.

Exclusion criteria were: a) being on different categories of antidepressants (e.g., venlafaxine, reboxetine, trazodone, bupropione); b) lifetime use of any kind of antipsychotics or mood stabilizers; c) being hospitalized primarily because of a comorbid diagnosis of psychiatric Axis I disorders; d) use of specific pharmacotherapy because of organic comorbidities (e.g., diabetes, epilepsy, inflammatory disorders).

According to international guidelines [35] during hospitalization all patients underwent the same multimodal intervention. Individualized treatment plans were managed by a multidisciplinary team composed by psychiatrists, clinical psychologists, registered dietitians, nurses and physicians trained in internal medicine. In more detail, the team was staffed by two psychiatrists with substantial experience in the treatment of AN; one of them (G.A.D.) had treated patients with AN for greater than 20 years and another (N.D.) for 10 years. In addition, the Program Director (S.F.) actively participated in clinical decision making. Given patients’ clinical severity upon admittance, the management of medical instability and nutritional rehabilitation had to be prioritized during the first days of treatment. After achieving medical stability, patients were then provided with daily individual motivational and psychotherapy sessions, and weekly psycho-educational groups to engage them as much as possible in the recovery process. All inpatients received five structured meals during the day under a dietitian’s supervision and enteral or parenteral feeding could be indicated as well based on clinical judgment. Blood tests and EKG were performed as frequently as needed by patients’ medical condition.

Given the retrospective design of this study, written informed consent was not required. Patients' records/information was anonymized and de-identified prior to analysis. The Ethics Committee of the Department of Neuroscience of the University of Turin approved this study.

### Measures

Patients’ height and weight were measured by a nurse at admission (T0) and discharge (T1) to calculate Body Mass Index (BMI). At the same time points, clinical interviews were performed by a psychiatrist to measure the weekly frequency of binge-purging behaviors (including use of diuretics and/or laxatives) and physical exercise. The following semi-structured interviews were also performed at both T0 and T1:

- The Hamilton Rating Scale for Anxiety (HAM-A) [36] is a clinician-administered scale of anxiety severity. Scores range from 0 to 56 (0–7: no or minimal symptoms, 8–17: mild anxiety; 18–24: moderate anxiety; and 25–30: severe anxiety). It showed good reliability, validity and sensitivity to change [37].
- The Hamilton Rating Scale for Depression (HAM-D) [38] is an interview with good psychometric properties [39] which has to be administered by a health care professional. It assesses depression through 21 questions. The higher the score, the more severe the depression as follows: 0–7 no depression; 8–13 mild depression; 14–18 moderate depression; 19–22 severe depression; ≥ 23 very severe depression.
- The Yale-Brown-Cornell Eating Disorders Scale (YBC-EDS) [40] is a clinician-rated interview with solid psychometrics that investigates core preoccupations and rituals related to eating disorders. Individual target symptoms are determined and then assessed in terms of time occupied by symptoms, interference with functioning, distress, and degree of control over symptoms. Four questions address preoccupations and four investigate rituals. Each item is rated on a scale anchored at 0 (none/not present) and 4 (extreme). All eight questions are added for the YBC-EDS total score and two YBC-EDS subtotal scores are also obtained. For the purpose of this study (i.e., measuring obsessive-compulsive traits) the motivation subscale was not included in the analysis.

### Statistical analysis

The Statistical Package for Social Sciences 21.0 (SPSS, SPSS Inc., Chicago, IL) was used for all analyses. A two-tailed alpha level of 0.05 was used for all statistical analyses.

Fisher’s exact test and one-way analysis of variance (ANOVA) with Bonferroni adjustments for multiple comparisons were used to analyze categorical and continuous variables, respectively, at baseline among the three groups.

Repeated-measures ANOVA with Bonferroni adjustments for multiple comparisons for the between-subject analysis was then used to assess changes on all variables considered between hospital admission and discharge.

The effect size (i.e., proportion of the variance in the dependent variable that can be explained by the independent one) of all findings was measured with the Partial Eta Squared (η_P_^2^). According to Cohen’s work [41] the effect size can be assessed as small η_P_^2^ = 0.01–0.06; moderate η_P_^2^ = 0.06–0.14; or large η_P_^2^>0.14.

## Results

### Clinical features of the sample

A total of 187 charts were overall reviewed (i.e., 100% of those admitted between January 2012 and May 2014). One-hundred and twelve had to be excluded because they failed to meet the inclusion criteria adopted for this study. Eighty-seven charts were discarded because of medications upon admission (39 on different antidepressants and 48 already on AAs); 19 due to a comorbid diagnosis which required patients’ hospitalization; and 6 because of organic disorders. Therefore, the charts of 75 patients were considered for this study. As shown in Table 1, the sample was composed by adult individuals (25.20±7.62 years) affected by severe (BMI: 13.93±1.93) and frequently enduring (6.85±6.10 years) AN. Participants were mostly women (n = 68, 90.7%).

**Table 1:** Clinical features of the sample and baseline differences between treatment groups.

	Total sample	Groups of treatment			Test statistics
	(n = 75,100%)	SSRIs (n = 25,32.9%)	Ari + SSRIs (n = 23,31.6%)	Ola + SSRIs (n = 27,35.5%)	p
Women, N(%)	68(90.7)	24(96)	22(95.6)	22(81.5)	0.205
Age, years, Mean(SD)	25.43(9.4)	25.2(7.62)	26.19(11.82)	24.96(8.81)	0.887
Duration of illness, years, Mean(SD)	6.85(6.12)	7.52(6.11)	6.29(6.92)	6.7(5.73)	0.785
AN subtype, N(%)					0.109
AN-R	50(66.7)	15(60)	13(56.5)	22(81.5)	
AN-BP	25(33.3)	10(40)	10(43.5)	5(18.5)	
BMI, Mean(SD)	13.93(1.93)	14.11(1.94)	14.11(2.11)	13.57(1.82)	0.5
Binging, Mean(SD)	2.21(5.61)	1.22(3.15)	4.65(8.33)	1.07(3.73)	**0.041** ^**a**^
Purging, Mean(SD)	3.13(6.26)	3.12(5.62)	5.78(8.71)	0.77(2.81)	**0.017** ^**b**^
Exercise, Mean(SD)	2.68(2.35)	2.52(2.32)	2.11(2.21)	3.33(2.41)	0.170
Diuretic abuse, N(%)	7(9.3)	3(12)	2(8.6)	2(7.4)	0.888
Laxative abuse, N(%)	7(9.3)	2(8)	5(21.7)	0(0)	**0.020** ^**c**^
HAM-A	20.88(7.84)	16.12(5.22)	23.52(7.21)	23.11(8.56)	**0.001** ^**d**^
HAM-D	23.03(7.64)	17.88(4.62)	26.48(6.73)	24.92(8.31)	**<0.001** ^**d**^
YBC-EDS T	18.82(4.38)	17.63(3.93)	18.84(5.15)	19.95(3.89)	0.170
YBC-EDS P	9.53(2.06)	8.85(1.84)	9.68(2.29)	10.06(1.95)	0.101
YBC-EDS R	9.28(2.58)	8.78(2.23)	9.16(3.07)	9.88(2.39)	0.306

Fisher’s exact test and one-way Analysis of Variance (ANOVA) with Bonferroni correction for multiple comparisons were used to analyze categorical and numerical variables, respectively.

^a^ significance did not hold after Bonferroni correction;

^b^ Ola+SSRIs<Ari+SSRIs; SSRIs = Ari+SSRIs, Ola+SSRIs;

^c^ Ari+SSRIs >Ola+SSRIs; SSRIs = Ari+SSRIs, Ola+SSRIs;

^d^SSRIs<Ari+SSRIs, Ola+SSRIs; Ari+SSRIs = Ola+SSRIs

Legend: SSRIs: selective serotonin reuptake inhibitors; Ari: aripiprazole; Ola: olanzapine; BMI: body mass index; AN: anorexia nervosa; AN-R: anorexia nervosa restricting subtype; AN-BP: anorexia nervosa binge-purging subtype; Binging: weekly binging episodes; Purging: weekly purging episodes; Exercise: weekly hours of exercise; HAM-A: Hamilton Rating Scale for Anxiety; HAM-D: Hamilton Rating Scale for Depression; YBC-EDS T: Yale-Brown-Cornell Eating Disorders Scale—total score; YBC-EDS P: Yale-Brown-Cornell Eating Disorders Scale—preoccupation subscale; YBC-EDS R: Yale-Brown-Cornell Eating Disorders Scale—rituals subscale.

All patients were on SSRIs (either sertraline or citalopram or escitalopram or fluoxetine) upon admission. Given the retrospective design of this study, medications were managed per clinical decision during the hospital stay and resulted distributed as follows: 25 individuals (32.9%) remained on SSRIs as monotherapy (eventually increasing the dosage; SSRIs), 23 (31.6%) started aripiprazole in addition to SSRIs (ARI), and 27 (35.5%) olanzapine in addition to SSRIs (OLA).

The three subgroups did not differ as regards age, gender, BMI, AN subtype, duration of illness, daily hours of exercise, and abuse of diuretics; however, as shown in detail in Table 1, differences emerged with respect to weekly binge-purging episodes and abuse of laxatives.

Mean duration of hospitalization was 34.78±9.85 days and did not differ among groups (SSIRs 34.52±6.17; ARI 32.67±10.97; OLA 36.55±11.59, F(2,72) = 0.93, p = 0.4). Mean duration of medication treatment was 4.96±1.62 weeks, mean olanzapine and aripiprazole doses were 6.11±3.27 and 9.13±6.33 mg/day, respectively. Occurrence of mild adverse events did not require discontinuation and was in line with previous literature [42]. The three groups did not differ in SSRIs composition or dosage (data not shown). The SSRIs group was composed by patients receiving: sertraline (T0: 87.50±32.73 mg/day; T1: 106.25±29.12), citalopram (T0: 23.33±5.16 mg/day; T1: 33.33±8.16), escitalopram (T0: 11±2.23 mg/day; T1:15±5), and fluoxetine (T0: 23.33±5.16 mg/day; T1: 36.66±5.16 mg/day). For those who received SSRIs as monotherapy, no switch to another antidepressant medication was performed. In case of increase of the dosage, all individuals started the higher dose during the first week of inpatient treatment.

### Changes in clinical variables amongst groups of treatment

Considering the overall sample, significant changes with large effect sizes emerged as regards all parameters considered (i.e., BMI, weekly binge-purging episodes and exercise, see Table 2).

**Table 2:** Changes in clinical variables between admission (T0) and discharge (T1) amongst groups of treatment.

	Total	SSRIs	Ari + SSRIs	Ola + SSRIs				Test statistics					
	(n = 75,100%)	(n = 25,32.9%)	(n = 23,31.6%)	(n = 27,35.5%)									
					Main effect of time			Main effect of group			Group-by-time interaction		
	Mean(SD)	Mean(SD)	Mean(SD)	Mean(SD)	F(1,72)	p	η_P_^2^	F(2,72)	p	η_P_^2^	F(2,72)	p	η_P_^2^
BMI					57.47	**<0.001**	0.44	0.63	0.533	0.02	0.27	0.763	0.007
T0	13.93(1.93)	14.11(1.94)	14.15(2.12)	13.57(1.81)									
T1	15.01(1.63)	15.06(1.47)	15.21(1.91)	14.77(1.55)									
Binging					13.04	**0.001**	0.15	3.49	**0.036** ^**a**^	0.09	2.63	**0.079**	0.07
T0	2.21(5.61)	1.22(3.15)	4.65(8.33)	1.13(3.73)									
T1	0.41(1.83)	0.21(0.41)	1.04(3.22)	0.04(0.19)									
Purging					17.95	**<0.001**	0.2	3.63	**0.032** ^**b**^	0.09	3.95	**0.024**	0.1
T0	3.11(6.26)	3.12(5.62)	5.78(8.61)	0.78(2.82)									
T1	0.61(2.69)	0.92(2.37)	0.87(4.17)	0.07(0.38)									
Exercise					85.88	**<0.001**	0.54	2.19	0.12	0.06	3.48	**0.036**	0.09
T0	2.68(2.35)	2.52(2.32)	2.12(2.21)	3.33(2.41)									
T1	0.71(0.93)	1.22(1.15)	0.21(0.42)	0.68(0.82)									

Repeated-measures analysis of variance (ANOVA) with Bonferroni adjustments for multiple comparisons was conducted.

^a^ Ari+SSRIs>Ola+SSRIs (trend towards significance after Bonferroni correction, p = 0.057); Ari+SSRIs >SSRIs (trend towards significance after Bonferroni correction, p = 0.09); SSRIs = Ola+SSRIs;

^b^ Ari+SSRIs >Ola+SSRIs; SSRIs = Ari+SSRIs, Ola+SSRIs.

Legend: SSRIs: selective serotonin reuptake inhibitors; Ari: aripiprazole; Ola: olanzapine; BMI: body mass index; Binging: weekly binging episodes; Purging: weekly purging episodes; Exercise: weekly hours of exercise.

η_P_^2^ = Partial Eta Squared; Cohen’s effect size: 0.01–0.06 = small effect; 0.06–0.14 = moderate effect; >0.14 = large effect.

As shown in Table 2, amongst the three groups no significant differences emerged with respect to changes in BMI and daily hours of exercise. In contrast, ARI when compared to OLA resulted significantly more effective in reducing purging episodes with a moderate effect size whilst as regards binging episodes after Bonferroni correction a trend towards significance emerged for ARI more effective than SSRIs (p = 0.09) and OLA (p = 0.057).

### Changes in general and eating psychopathology amongst groups of treatment

Considering the sample as a whole, significant changes with large effect sizes emerged on all semi-structured interviews considered (i.e., HAM-A, HAM-D, YBC-EDS total score and both subscales) comparing T0 and T1 scores.

**Fig 1:**
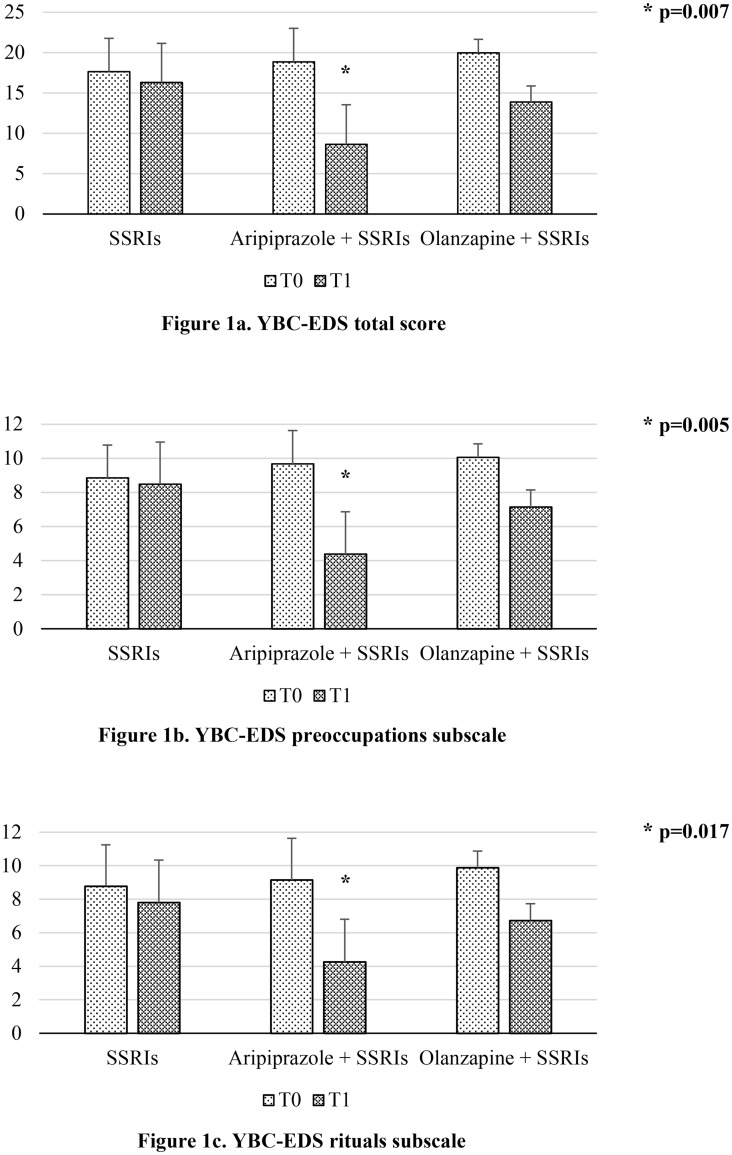
Changes in Yale-Brown-Cornell Eating Disorders Scale (YBC-EDS) total score (1a) and preoccupations (1b) and rituals (1c) subscales amongst groups of treatment at admission (T0) and discharge (T1). All groups significantly improved on all YBC-EDS scales but those patients undergoing aripiprazole in combination with Selective Serotonin Reuptake Inhibitors (SSRIs) reported a significantly larger improvement when compared to the other groups. Mean scores and 95% confidence intervals are shown as columns and bars, respectively.

As shown in Table 3, at the two time-points considered the three groups did not differ significantly from one another as regards anxiety (i.e., HAM-A). In contrast, differences on depression (i.e., HAM-D) and eating-related rituals and preoccupations (i.e., YBC-EDS total score and both preoccupations and rituals subscales) emerged. As shown in Table 3, OLA showed a trend towards a greater effect than SSRIs on depression. With respect to eating preoccupations and rituals, ARI reported a significantly greater improvement with a large effect size on the global score and both subscales of the YBC-EDS when compared to both other groups (Fig 1).

**Table 3:** Changes in anxiety, depression, social anxiety and eating preoccupations/rituals as measured with semi-structured interviews between admission (T0) and discharge (T1) amongst groups of treatment.

	Total	SSRIs	Ari + SSRIs	Ola + SSRIs				Test statistics					
	(n = 75,100%)	(n = 25,32.9%)	(n = 23,31.6%)	(n = 27,35.5%)									
					Main effect of time			Main effect of group			Group-by-time interaction		
	Mean(SD)	Mean(SD)	Mean(SD)	Mean(SD)	F(1,72)	p	η_P_^2^	F(2,72)	p	η_P_^2^	F(2,72)	p	η_P_^2^
HAM-A					52.89	**<0.001**	0.42	2.95	0.059	0.77	11.38	**<0.001**	0.24
T0	20.87(7.84)	16.12(5.22)	23.52(7.21)	23.11(8.56)									
T1	13.43(7.04)	14.92(4.88)	10(6.16)	15.04(8.52)									
HAM-D					66.09	**<0.001**	0.48	3.15	**0.049** ^**a**^	0.08	10.77	**<0.001**	0.23
T0	23.03(7.64)	17.88(4.61)	26.48(6.73)	24.92(8.31)									
T1	14.36(6.43)	15.82(4.26)	12.17(7.63)	14.92(6.74)									
YBC-EDS T					86.01	**<0.001**	0.55	5.39	**0.007** ^**b**^	0.13	15.96	**<0.001**	0.31
T0	18.82(4.38)	17.63(3.93)	18.84(5.15)	19.95(3.89)									
T1	13.06(5.93)	16.29(3.76)	8.64(4.63)	13.87(6.42)									
YBC-EDS P					80.63	**<0.001**	0.53	5.75	**0.005** ^**b**^	0.14	19.41	**<0.001**	0.35
T0	9.53(2.06)	8.85(1.84)	9.68(2.29)	10.06(1.95)									
T1	6.73(3.06)	8.48(2.01)	4.38(2.35)	7.14(3.22)									
YBC-EDS R					75.97	**<0.001**	0.52	4.32	**0.017** ^**b**^	0.11	10.63	**<0.001**	0.23
T0	9.28(2.58)	8.78(2.23)	9.16(3.07)	9.88(2.39)									
T1	6.33(2.99)	7.81(2.01)	4.26(2.41)	6.73(3.31)									

Repeated-measures analysis of variance (ANOVA) with Bonferroni adjustments for multiple comparisons was conducted.

^a^ Ola+SSRIs >SSRIs (trend towards significance after Bonferroni correction, p = 0.057); SSRIs = Ari+SSRIs; Ari+SSRIs = Ola+SSRIs.

^b^ Ari+SSRIs >SSRIs, Ola+SSRIs; SSRIs = Ola+SSRIs.

Legend: SSRIs: selective serotonin reuptake inhibitors; Ari: aripiprazole; Ola: olanzapine; HAM-A: Hamilton Rating Scale for Anxiety; HAM-D: Hamilton Rating Scale for Depression; YBC-EDS T: Yale-Brown-Cornell Eating Disorders Scale—total score; YBC-EDS P: Yale-Brown-Cornell Eating Disorders Scale—preoccupation subscale; YBC-EDS R: Yale-Brown-Cornell Eating Disorders Scale—rituals subscale.

η_P_^2^ = Partial Eta Squared; Cohen’s effect size: 0.01–0.06 = small effect; 0.06–0.14 = moderate effect; >0.14 = large effect.

## Discussion

No medications are currently indicated for the treatment of AN [6]. With this study we aimed to garner preliminary real-world data on the management of the combined use of pharmacological agents in AN with the overarching aim to stimulate the debate on this topic. All in all, we found encouraging data on the use of aripiprazole as augmentation agent to reduce eating-related preoccupations and rituals although caution is needed when interpreting these findings.

In fact, it should be borne in mind that those patients who were started with an augmentation agent were characterized on average by greater clinical severity upon admission than those who received SSRIs as monotherapy, as expected according to the retrospective design of this study. Still, in order to make the sample as clean as possible, many patients (n = 112) had to be excluded according to the aforementioned criteria. Taken together, these elements may represent a selection bias. This said, we believe these data could represent a useful, although preliminary, insight into a neglected topic, namely the use of AAs as augmentation agents in AN. Still, in line with the exploratory aim of this work, to the best of the authors’ knowledge, only very little data [17,43] is currently available on the use of aripiprazole in AN.

The relevance of clinical judgment has been also recently highlighted [9] and in this study, per clinical decision, those with a greater number of weekly binge-purging episodes were found to have been started with aripiprazole. This choice was made by our group in light of the hunger-neutrality of this medication; therefore, it was most often used in order to avoid exacerbating binge-purging behaviors. In contrast, those with severe physical hyperactivity were started with olanzapine because of its beneficial effects on anxiety, agitation, and aggressiveness also due to its sedative properties [8,19]. Given the greater number of weekly binge-purging episodes in the ARI group when compared to OLA, it is unwise to draw firm conclusions on the effectiveness of aripiprazole in reducing binge-purging episodes. Although intriguing, future studies on a more homogeneous sample are warranted to shed light on this issue.

Differently from what initially hypothesized, BMI significantly increased at discharge with no differences amongst groups. Although olanzapine-induced weight gain in AN has been questioned [11,12,19], such a compound has been found to play a role in this regard [20]. However, since the three groups had comparable BMI upon admittance, the lack of differences amongst groups is of interest and highlights the role of the multimodal approach adopted during hospitalization instead of the relevance of medications *per se*.

The main finding of this work is represented by the positive effects with large effect size we found with the adjunct of aripiprazole to antidepressants on eating-related obsessive-compulsive symptomatology. Notwithstanding comparable baseline scores, the ARI group showed a significantly more marked improvement on YBC-EDS global score and both subscales when compared to the others. Obsessive-compulsive traits are frequently intertwined with AN since they not only characterize the course of illness but also tend to predate its onset [31]. Still, the lifetime prevalence of OCD is elevated in AN [21]. Moreover, this improvement is of interest since it has been found that obsessive-compulsive traits adversely affect outcome [44] and it is in line with what Trunko and collaborators [17] described in their case report. In fact, the latter group described how aripiprazole contributed to improve several psychopathological aspects of AN (e.g., distress around eating, food and weight-related obsessional thoughts) and comorbid conditions like depression and generalized anxiety [17]. Another interesting element is that obsessions have been recently related to purging behaviors [45] in AN and we consistently found aripiprazole as effective on both aspects.

Speculating on the potential mechanisms by which this medication might be effective is way beyond the scope of this paper. Nevertheless, it could be raised the hypothesis that its partial agonist action might be of some utility in modulating dopamine levels which are strictly related to anxiety in AN [4]. Also, it is not even possible to ascertain whether such an improvement is related to anxious/depressive comorbidity or to the effect of aripiprazole itself. Finally, patients with psychotic symptoms [46] or at least AN-related delusional aspects might have benefited from the antipsychotic properties of aripiprazole. In fact, it is noteworthy that psychotic features occur in 1 out 5 cases of AN with delusional thinking correlating with drive-for-thinness but not with other indexes of illness severity, including BMI [47]. Given the potential clinical implications of this finding, further studies may want to better investigate such encouraging data.

With respect to general psychopathology, both augmentation agents did not result as more effective than SSRIs alone in reducing anxiety; olanzapine showed only a trend towards a more beneficial result on depression when compared to SSRIs. Again, the SSRIs group was significantly less anxious and depressed than ARI and OLA. Therefore, the use of AAs could be in line with treatment principles that are applied to other psychiatry disorders (e.g., severe depression and obsessive-compulsive disorders). In such cases, augmentation with AAs is a therapeutic option in case of scarce or only partial response to SSRIs and it may be of some help with comorbid conditions in AN as well. On the contrary, specific effects failed to emerge in this sample and our hypothesis was not confirmed in this regard although baseline differences should be borne in mind when interpreting these results. Still, our findings are only partially in line with previous studies reporting olanzapine to be effective on depression and anxiety [10,19,48]. Nevertheless, previous work focused on outpatients and olanzapine was not used as an augmentation agent; this is of importance since SSRIs could modify the antidepressant effect of olanzapine in monotherapy. On the other hand, the dearth of clinical studies on aripiprazole makes it difficult to compare our data with the current literature.

Some limitations should be acknowledged. First, a retrospective design was adopted and the groups were not randomized; as a result, clinical severity was not homogeneous across groups. Relatedly, some differences with respect to eating symptomatology emerged. Still, no self-report instruments were adopted and both time and dose were flexible. Moreover, length of treatment may impact medication effects; namely, the effects of AAs could be overestimated given their more rapid action when compared to SSRIs. Finally, olanzapine and aripiprazole were slightly unmatched as regards equivalent doses; however, the latter were established according to the literature on the spectrum of psychotic disorders [49] so differences may exist for AN.

In closing, we found aripiprazole to be mostly effective in reducing eating psychopathology, namely eating-related preoccupations and rituals, on a sample of inpatients diagnosed with AN. Aripiprazole did not differ instead from either SSRIs as stand-alone therapy or olanzapine as augmentation agent of SSRIs in either weight gain or improvement of eating symptomatology and general psychopathology. Future double blind randomized controlled trials are warranted to confirm these novel and promising preliminary data on the use of augmentation strategies in AN. Moreover, as recently proposed [9], research should also endeavor to identify what subgroups of patients could benefit more from AA augmentation.
